# The Clinical Utility of Precision-Guided Dosing for Adalimumab Therapy Optimization in Inflammatory Bowel Disease: A Clinical Experience Program †

**DOI:** 10.3390/pharmaceutics17040428

**Published:** 2025-03-27

**Authors:** Stephen B. Hanauer, Esther A. Torres, Patricia Aragon-Han, Jonathon C. Chapman, Arun C. Swaminath, Ronen Arai, Mandalina Butnariu, Thomas C. Lee, Shervin Rabizadeh, Morgan Check, Terrence A. Barrett, Jana G. Hashash, Thomas Meister, Eugene F. Yen, Jami Kinnucan, Daniel J. Stein, David Ziring, Rimma Shaposhnikov, Preetika Sinh, Taha M. Qazi, Andres J. Yarur, Farah Monzur, Thierry Dervieux, Bincy P. Abraham

**Affiliations:** 1 School of Medicine, Northwestern University Feinberg, Chicago, IL 60611, USA; shanauer@northwestern.edu (S.B.H.); eugene.yen@nm.org (E.F.Y.); 2 Department of Medicine, School of Medicine, University of Puerto Rico Medical Sciences Campus, San Juan 00936, Puerto Rico; estheratorresmd@gmail.com; 3 Prometheus Laboratories Inc., San Diego, CA 92121, USA; paragonhan@prometheuslabs.com; 4 Baton Rouge General Medical Center—Mid City, Baton Rouge, LA 70806, USA; jchapman@tddctx.com; 5 Lenox Hill Hospital, New York, NY 10075, USA; aswaminath@northwell.edu; 6 GastroHealth, Coral Springs, FL 33065, USA; rarai@gastrohealth.com; 7 Division of Gastroenterology, The Ohio State University Wexner Medical Center, Columbus, OH 43210, USA; madalina.butnariu@osumc.edu; 8 Associated Gastroenterologists of CNY, Camillus, NY 13031, USA; drthomaslee@gmail.com; 9 Cedars-Sinai Medical Center, Los Angeles, CA 90048, USA; shervin.rabizadeh@cshs.org (S.R.); morgan.check@cshs.org (M.C.); david.ziring@cshs.org (D.Z.); andres.yarur@cshs.org (A.J.Y.); 10 Division of Digestive Disease and Nutrition, University of Kentucky Medical Center, Lexington, KY 40536, USA; t.barrett@uky.edu; 11 Mayo Clinic, Florida, Jacksonville, FL 32224, USA; alhashash.jana@mayo.edu (J.G.H.); kinnucan.jami@mayo.edu (J.K.); 12 Gastroenterology Associates Colorado Springs, Colorado Springs, CO 80907, USA; tomsbulk@hotmail.com; 13 Division of Gastroenterology, Medical College of Wisconsin, Milwaukee, WI 53226, USA; dstein@mcw.edu (D.J.S.); psinh@mcw.edu (P.S.); 14 UCLA Westlake Village, Thousand Oaks, CA 91362, USA; rshaposhnikov@mednet.ucla.edu; 15 Cleveland Clinic, Cleveland, OH 44195, USA; qazit@ccf.org; 16 Stony Brook Medicine, Stony Brook, NY 11794, USA; farah.monzur@stonybrookmedicine.edu; 17 Houston Methodist, Houston, TX 77030, USA; bpabraham@houstonmethodist.org

**Keywords:** precision-guided dosing, precision medicine, dose optimization, real-world data, clearance, adalimumab, pharmacokinetics, therapeutic drug monitoring, inflammatory bowel disease

## Abstract

**Background/Objectives**: This study aimed to establish the clinical utility of a therapeutic drug monitoring (TDM)-supported, model-informed precision dosing (MIPD) approach (precision-guided dosing [PGD]) by assessing the impact of pharmacokinetic (clearance [CL]) and clinical laboratory parameters on adalimumab (ADA) dosage adjustments during maintenance therapy for inflammatory bowel disease (IBD). **Methods**: In the EMPOWER study, blood was collected at any time post-ADA injection. Pharmacokinetic (PK) testing was conducted in an accredited lab. Inputs for the PGD test included ADA concentrations, antibodies to ADA, albumin levels, and the current dosing regimen. CL was calculated using nonlinear mixed-effect models. Results were reported to health care providers (HCPs) within 3 days. HCPs’ treatment decisions were recorded and classified as treatment reduction, continuation, intensification, or ADA discontinuation. The physician global assessment (PGA) of disease activity was collected. Relationships between drug concentrations, CL, disease activity, and physician decision-making were assessed using logistic regression. **Results**: A total of 213 cases were assessed by 21 HCPs. ADA treatment was intensified in 24% and discontinued in 13% of cases. An ADA concentration ≤ 10 μg/mL was associated with a 23.7-fold and 3.0-fold higher likelihood of therapy intensification and PGA > 0, respectively, compared to concentrations > 10 μg/mL. An ADA concentration < 5 μg/mL was associated with a 3.3-fold higher likelihood of treatment discontinuation. CL ≥ 0.318 L/day was associated with a 10.4-fold higher likelihood of therapy intensification. Higher CL (>0.8 L/day) was associated with a 3.5-fold and 4.2-fold higher likelihood of treatment discontinuation and PGA > 0, respectively. **Conclusions**: PGD enables earlier and precise optimization of ADA dosing by predicting trough levels at any time during the therapy cycle. Optimized dosing to achieve target ADA concentrations and low clearance is crucial to mitigate therapy discontinuation and active disease in IBD patients.

## 1. Introduction

Crohn’s disease (CD) and ulcerative colitis (UC), collectively termed inflammatory bowel disease (IBD), affect between 2.4 and 2.7 million people in the US [1]. Notably, up to 25% of patients are diagnosed before the age of 20 years [2,3].

IBD can lead to complications, surgery, and disability [4]. Pharmacological treatments include anti-inflammatory compounds with various mechanisms of action. Recent studies based on claims data have shown that approximately 80% of patients had at least one indicator of suboptimal therapy [5,6]. Not surprisingly, health care costs were higher in both CD and UC when suboptimal therapy indicators were present, and costs increased as the number of indicators increased [5,6], highlighting the financial burden of unoptimized therapy and unmanaged disease [6,7,8,9].

Several studies have demonstrated that patients who achieve mucosal healing experience fewer complications, hospitalizations, and intestinal resections compared to patients who do not, making mucosal healing an important treatment goal in the treat-to-target approach in IBD [10,11,12]. Mucosal healing as an endpoint for decision-making has also been shown to be cost effective [10].

Anti-tumor necrosis factor alpha (anti-TNFα) is more efficacious than conventional treatments for inducing and maintaining clinical remission and mucosal healing, and for reducing corticosteroid use, surgeries, and hospitalizations in CD and UC [13,14]. However, approximately 30% of patients do not respond adequately to anti-TNFα therapy, and 40–50% lose response over time [13,15,16,17]. Primary and secondary loss of response to biologics may be due to mechanistic failure or to subtherapeutic drug concentrations in the presence or absence of anti-drug antibodies. Trough concentrations can vary from individual to individual, due to characteristics such as sex, body mass index, inflammatory burden, disease activity, the presence of anti-drug antibodies, and drug clearance (CL) [18,19].

Numerous studies have shown positive associations between concentrations of anti-TNFα and other biologics, with clinical response, remission, mucosal healing, and lower C-reactive protein (CRP) and fecal calprotectin [15,16,18,20,21], supporting the role of therapeutic drug monitoring (TDM) in the treat-to-target paradigm. While reactive TDM is performed in the setting of loss of response or intolerance to a monoclonal antibody, proactive TDM may prevent loss of response [20]. The latter approach has been associated with better clinical outcomes, reduced surgeries and hospitalizations, and, therefore, lower health care costs, compared to reactive TDM [20,22,23,24], possibly because it allows for the adjustment of dosing to maintain an appropriate drug concentration [25]. In addition, the use of multiple predictive factors of pharmacokinetic (PK) origin, such as concentration and CL in combination, leads to superior disease control and remission [26].

Drug CL, which is the volume of serum from which a drug is removed from the body as a function of time (expressed as liters per day), has a major impact on drug concentrations. In IBD, CL of anti-TNFα is influenced by several factors, including the presence of anti-drug antibodies, intrinsic metabolism and recirculation of the drug, inflammatory burden, protein loss through inflamed mucosa, and other patient characteristics [27,28]. As high infliximab (IFX) CL in IBD is associated with worse outcomes [26,27,29], it is important to consider CL when optimizing therapy in individual patients.

Precision-guided dosing (PGD) is a TDM-supported, model-informed precision dosing (MIPD) approach designed for two of the most commonly used anti-TNFα in IBD, IFX and adalimumab (ADA) [30], or their biosimilars. Developed and validated by Prometheus Laboratories Inc., PGD incorporates population PK modeling with Bayesian priors and patient-specific clinical and serological inputs to provide individualized PK parameters, as well as alternative dosing strategies [31,32]. In Wright et al. (2023) [32], endoscopic remission, clinical remission, and fecal calprotectin < 100 µg/g were shown to be associated with ADA CL < 0.318 L/day, with CL having better performance than drug concentration. In addition, in a real-world clinical utility study, PGD for IFX impacted treatment decisions by enabling earlier and more precise dose optimization of IFX in patients with IBD, possibly leading to improved health outcomes and overall cost savings [33,34].

The objective of this clinical experience program (CEP) was to evaluate the real-world clinical utility [35] of PGD by evaluating how health care providers (HCPs) use this test to guide ADA dosage adjustments, through the integration of pharmacokinetics—particularly CL—and clinical laboratory parameters. By incorporating these factors, PGD provides a precision-based approach to support therapy decisions for patients with IBD on ADA maintenance.

## 2. Materials and Methods

### 2.1. Precision-Guided Dosing for ADA

PGD for ADA is a TDM-supported MIPD tool designed to optimize dose and injection frequency during the maintenance phase (≥8 weeks of therapy) of ADA or its biosimilars in adult patients with IBD. All ADA concentrations used in this study were measured in the maintenance phase, to ensure that dose adjustments were based on steady-state pharmacokinetics, rather than induction-phase variability. Blood samples could be collected at any time post-injection.

ADA serum concentration (lower limit of quantification: 1.6 µg/mL), anti-drug antibody (lower limit of quantification: 1.7 U/mL), and albumin (g/dL), were measured in a CLIA-certified, CAP-accredited laboratory (Prometheus Laboratories Inc., San Diego, CA, USA), as previously described [32]. These measured values, along with the patient’s ADA dose, dosing frequency, and time since last injection, were incorporated into a validated Bayesian forecasting model.

ADA CL was estimated using a validated population pharmacokinetic (PopPK) model with Bayesian forecasting [32]. A one-compartment model with first-order absorption and elimination was developed (Monolix 5.1.0; Lixoft, Antony, France), using a nonlinear mixed-effects approach. CL was individually estimated, while the volume of distribution (V/F = 8.9 L) and absorption rate (ka = 0.2 L/day) were fixed based on prior studies [32]. Anti-drug antibody and serum albumin parameters were included as key covariates influencing CL. Model performance was assessed using goodness-of-fit tests and predictive checks. Bayesian priors estimated individual CL and predicted ADA trough levels at 7 or 14 days post-dose.

The PGD test report included estimated CL (L/day), measured ADA concentrations (µg/mL), level of anti-drug antibody (U/mL), albumin levels (g/dL), and predicted trough ADA concentrations at different dosing regimens, specific to each patient’s PK profile. Results were reported to the ordering HCP within three days of sample receipt, allowing real-time integration into treatment decisions.

### 2.2. Clinical Experience Program (CEP)

The EMPOWER ADA (Effect on Decision-Making of Precision Optimization in Real-World Evidence Research—Adalimumab) study was a prospective, cross-sectional, multicenter, survey-based CEP conducted from March 2023 to January 2024.

Similarly to the EMPOWER IFX CEP [33], the HCPs who agreed to participate completed a pre-program survey on their typical use of TDM in practice. HCPs were allowed to use up to 20 PGD tests for patients aged 18–80 years with a confirmed diagnosis of IBD on ADA maintenance therapy (≥8 weeks of therapy), with or without concomitant immunomodulators or steroids. Pregnant patients were excluded from the study.

Selection bias was minimized as treating HCPs were allowed to order PGD tests at their discretion, based on SOC practice. The study did not impose additional selection criteria beyond routine clinical decision-making, ensuring a real-world representation of TDM utilization. Additionally, testing could be ordered for different patients or for the same patient over time (repeat testing), further broadening the study’s applicability.

Informed consent was waived for this study, due to its nature as a CEP, rather than an interventional trial (additional information can be found in the Institutional Review Board statement section). The PGD test was ordered as part of routine TDM under standard-of-care (SOC), with no additional interventions introduced beyond normal clinical practice. Importantly, no patient identifiers were collected, and data were analyzed in aggregate at the clinician level. The study focused on HCP decision-making, rather than direct patient-reported outcomes, and the test was ordered at the discretion of the treating HCP, mirroring routine TDM practices.

After receiving each PGD test result, the HCPs completed a post-test survey, capturing disease characteristics (including Montreal classification) [36], concomitant medications, prior biologic and/or advanced therapy use, reason(s) for ordering the test [37], how results were used to guide treatment, and disease activity. Disease activity was assessed using the physician global assessment (PGA): remission/no symptoms (PGA = 0); mild (PGA = 1); moderate (PGA = 2); severe (PGA = 3 or not assessed) [38,39]. Mild symptoms were characterized by occasional abdominal discomfort and fewer than four loose stools per day, without systemic symptoms, whereas moderate symptoms involved intermittent abdominal pain and four to six stools per day, with minimal systemic symptoms [38,39]. For each patient, post-test treatment decisions were classified as follows: reduction (decreased ADA dose and/or frequency); continuation (unchanged ADA dose and frequency); intensification (increased ADA dose and/or frequency); or discontinuation (switch to a different biologic or treatment). At the end of their participation in the program, the HCPs completed a post-program survey to provide overall feedback on the utility of the PGD test.

The survey questions were adapted from those used in the EMPOWER IFX CEP [33], incorporating insights from the expert consensus literature [37], previous data, and expert opinion. The logic was tested internally before distribution of the surveys to HCPs.

### 2.3. Data Analysis

Since forecasted trough concentrations and measured ADA concentrations throughout the cycle were very highly correlated (R^2^ = 0.9431, Deming’s slope = 0.9905) (Supplementary Figure S1), measured ADA concentrations were used for analysis.

Descriptive statistics summarized patient characteristics, while comparative analyses assessed differences across treatment decision groups (reduction, continuation, intensification, discontinuation) to identify factors associated with ADA therapy modifications. Kruskal–Wallis tests were applied for continuous and ordinal variables, and Fisher’s exact tests for categorical variables.

Univariate logistic regression was performed to estimate the likelihood of treatment adjustments (intensification, discontinuation) and active disease (PGA > 0) (dependent variables) based on ADA concentration and CL (independent variables). Multivariate analysis was not conducted to avoid overfitting, as PGD testing already integrates albumin, dose, and interval to estimate CL. Separate models were used for ADA concentration and CL, given their inherent dependency.

Multivariate logistic regression was performed to predict independent factors contributing to high CL, using backward elimination of variables based on *p*-values. PK cutoffs were based on the literature, where CL above 0.318 L/day and ADA concentrations below 10 µg/mL were associated with poor therapeutic outcomes in CD [32]. Disease was considered active if the patient was not in remission (PGA > 0).

Results were reported as odds ratios (ORs) and 95% confidence intervals (CIs) or interquartile ranges (IQRs). A *p*-value < 0.05 was used to determine statistical significance. Statistical analysis was performed using R 4.3.1 [40].

## 3. Results

### 3.1. Characteristics of Clinical Practices Participating in Program

A total of 22 HCPs specializing in IBD, from 12 academic and 10 community centers, accepted the invitation to participate in the program; all but one academic center completed post-test surveys. The 21 participating HCPs ordered 213 tests (2 to 20 tests per HCP) for a total of 207 unique adult patients, and all completed the corresponding 213 post-test surveys and the post-program questionnaire.

The participating HCPs were in 10 different locations in the United States and Puerto Rico. The majority (85.7%) had been in practice for 10 years or more, and all were highly experienced in treating patients with IBD: 6 out of 21 (28.6%) HCPs treated at least approximately 25 patients with IBD per month, while 14 (66.7%) treated more than 50 per month (Supplementary Table S1).

In the pre-program survey, 66.7% of HCPs indicated that they used ADA or a biosimilar in 10% to 20% of their patients. TDM was utilized in symptomatic patients (primary or secondary loss of response; reactive TDM), as well as to optimize ADA therapy in the absence of symptoms (proactive TDM) (Supplementary Figure S2). See Supplementary Table S1 for the complete pre-program survey results.

### 3.2. Patient Characteristics

The median age of the cases for whom a PGD test was ordered was 40 (range: 18–88) years; 49% were female, 76% had CD, 22% had UC, and 2% had indeterminate colitis (IC). Based on the PGA, 46% of cases were in remission (Table 1), whereas 34%, 14%, and 6% had mild, moderate, and severe disease activity, respectively.

### 3.3. Impact of PGD Test on HCP Treatment Decisions and Disease Activity

Overall, the median concentration of ADA was 12 µg/mL, and 6.6% (14/213) of samples had detectable anti-drug antibodies (Table 1). The median CL was 0.334 L/day, slightly above the cutoff of lower CL (0.318 L/day) associated with better outcomes [32].

**Figure 1:**
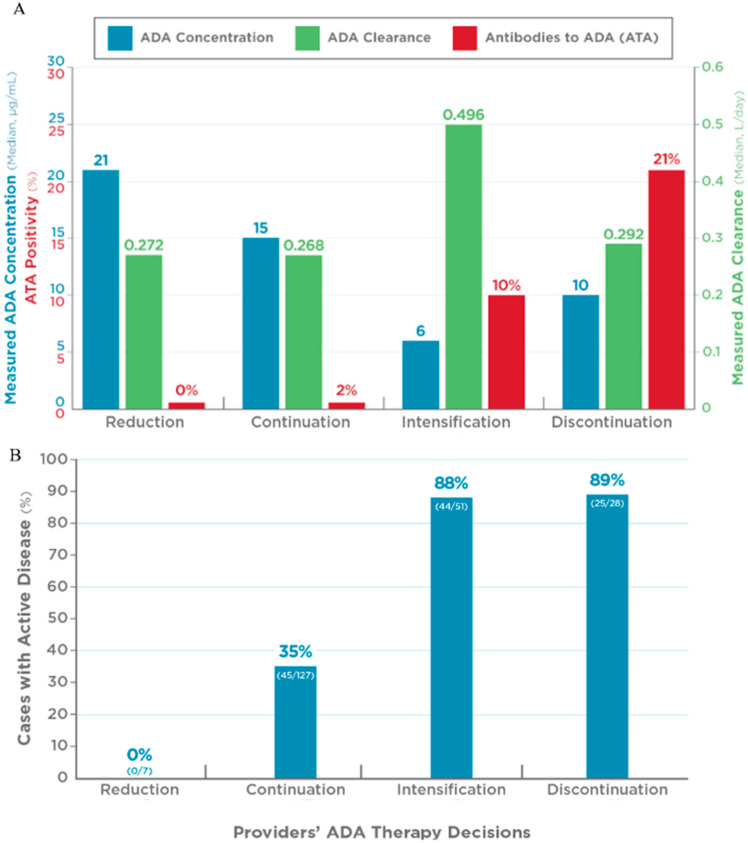
Relationship between precision-guided dosing (PGD) test results (measured ADA, presence of anti-drug antibodies, and CL) and physician global assessment (PGA) with changes in ADA therapy. (**A**). Median adalimumab (ADA) concentration and percentage of cases with detectable anti-drug antibodies in cases for whom ADA therapy was reduced (reduction), continued unchanged (continuation), intensified (intensification), or discontinued (discontinuation). (**B**). Percentage of cases with physician global assessment [PGA] > 0 (active disease) in each group.

Based on the post-test surveys, ADA treatment was reduced or remained unchanged for 7 (3%) and 127 (60%) of the cases, respectively. All the cases for whom therapy was reduced were in remission. Although 45 (35%) cases whose therapy remained unchanged were not in remission, 37 (82%) had mild disease (PGA = 1), and only 8 had a PGA of 2 or 3. The two groups for whom ADA treatment was reduced or remained unchanged had the highest median ADA concentrations (21 and 15 µg/mL, respectively) and the lowest median CL (0.272 and 0.268 L/day, respectively). In addition, the majority of cases in these groups had ADA concentrations > 10 µg/mL (86% and 80%, respectively) and CL < 0.318 L/day (71% and 58%, respectively; Table 1 and Figure 1).

Treatment with ADA was intensified for 51 (24%) and discontinued for 28 (13%) cases, consistent with the fact that most cases in these groups (88% and 89%, respectively) were not in remission. These two groups presented with the lowest ADA concentrations (median 6 and 10 µg/mL), the highest rate of anti-drug antibodies (9.8% and 21%), and the highest ADA CL (median 0.496 and 0.292 L/day, respectively). Consistently with these data, only 12% of cases in the intensification group presented with an ADA concentration > 10 µg/mL and CL < 0.318 L/day. In the discontinuation group, 57% of cases had an ADA concentration > 10 µg/mL, and 54% had CL < 0.318 L/day (Table 1 and Figure 1).

Of the 28 cases in the discontinuation group, 12 had an ADA concentration ≤ 10 µg/mL and 16 had a concentration > 10 µg/mL. In addition, anti-drug antibodies were detected in 6 of these 12 cases (50%), whereas none of the 16 cases with ADA > 10 µg/mL had anti-drug antibodies (*p* = 0.002). Other baseline characteristics, including PGA and albumin, were not statistically different between the two subgroups.

**Figure 2:**
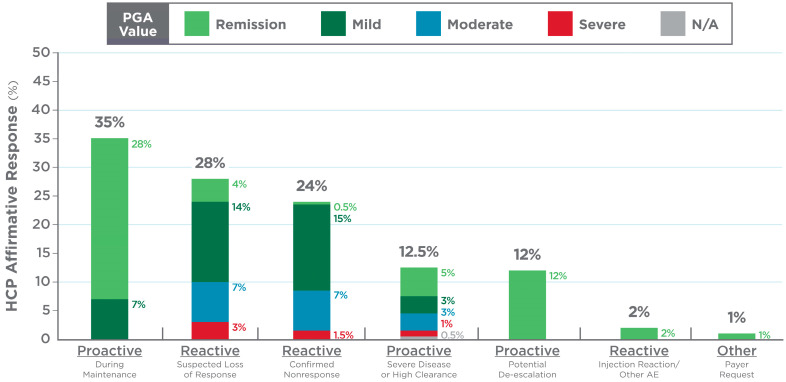
Reasons for ordering precision-guided dosing (PGD) test based on post-test survey. Bars report percentage of health care providers (HCPs) responding “yes” to reasons for using PGD test for particular patient. HCPs could select multiple reasons. Thus, total exceeds 100%. Proactive and reactive describe PGD test use. One case had no PGA (N/A). Abbreviations: AE (adverse event).

In the post-test survey, the HCPs recorded the reasons for ordering a PGD test in each case. In most cases, the test was ordered to support dose optimization or to guide therapy de-escalation when patients were in remission or had mild symptoms. Conversely, disease activity was higher if the test was ordered when patients were not responding to therapy or when loss of response was suspected (Figure 2).

We also evaluated whether other therapy changes were instituted, and whether imaging or laboratory tests were ordered. Immunomodulators were added in five cases (10%) in the intensification group, and were discontinued in the reduction and continuation groups (14% and 6%, respectively). Cases in the discontinuation group were switched from ADA to compounds with a different mechanism of action (Supplementary Table S3). Imaging and laboratory tests were ordered for a total of 23 and 8 cases, respectively, in the continuation, intensification, and discontinuation groups, whereas no imaging and laboratory tests were ordered for cases in the reduction group (Supplementary Table S3 and Supplementary Figure S3).

Logistic regression was used to analyze the relationship between ADA concentration or CL and therapy changes or disease activity. A measured concentration of ADA ≤ 10 µg/mL was associated with a 23.7-fold higher likelihood of ADA therapy intensification (*p* < 0.001) and with a 3.02-fold higher likelihood of PGA > 0, compared to ADA concentrations > 10 µg/mL (*p* < 0.001). In addition, ADA < 5 µ/mL was associated with ADA discontinuation (Table 2). The same analysis was conducted to evaluate the relationship between CL and ADA therapy changes or disease activity. CL ≥ 0.318 L/day was associated with a 10.4-fold higher likelihood of ADA therapy intensification (*p* < 0.001) compared to CL < 0.318 L/day. High CL (> 0.8 L/day) was associated with ADA discontinuation, and PGA > 0 (active disease) with a 3.49- and 4.20-fold higher likelihood (*p* = 0.002), respectively (Table 2).

### 3.4. Factors Associated with High CL

Given that several factors contribute to the variability of drug CL in IBD [27,28], we carried out a multivariate analysis to elucidate what factors were associated with high ADA CL (>0.8 L/day) in this patient population. As reported in Table 3, sex, age, diagnosis (CD or UC), disease duration, concomitant use of immunomodulators or steroids, and Montreal behavior were not associated with high CL. On the other hand, a statistically significant association was found with PGA > 0 (active disease), presence of anti-drug antibodies, albumin concentration, and Montreal location. After backward elimination of factors with *p* > 0.05, these factors remained significantly associated with high CL. The clinical factor most strongly associated with high CL was the presence of anti-drug antibodies (OR = 127, *p* < 0.001; Table 3), consistent with previous data [28].

### 3.5. Post-Program Survey

**Figure 3:**
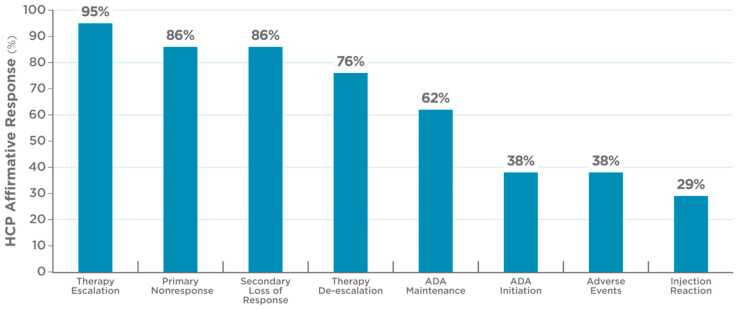
Usefulness of precision-guided dosing (PGD) test, as indicated in post-program survey. Bars report percentage of health care providers (HCPs) who answered “yes” to scenarios where they found PGD test most useful. HCPs could select multiple reasons. Thus, total exceeds 100%. ADA refers to adalimumab or its biosimilars.

All HCPs indicated that the test had high value in aiding precision-guided ADA dosing (Figure 3). In particular, 95% of HCPs reported that the test was useful for therapy escalation, 76% reported that it was useful for de-escalation, and 86% found the test useful for patients experiencing primary or secondary loss of response. Consistently with these data, 81% of the HCPs indicated that the PGD test assisted in deciding the optimal ADA dose and/or interval of administration, and 67% indicated that the test strengthened their confidence in the ongoing treatment regimen. The PGD test was also helpful in providing justification to insurance providers for adjustments of dose and/or interval (48%), or in discussions with patients regarding dose escalation (81%) and adverse events (29%). HCPs indicated that the test was easy to order (86%), the test result turnaround time was acceptable for impacting their clinical decision-making (95%), and the test report was easy to understand (100%). See Supplementary Table S2 for the complete results of the post-program questionnaire.

Eleven HCPs (52%) provided comments, including that the test was valuable, easy to use, and that it allowed early identification of patients needing ADA escalation or discontinuation.

## 4. Discussion

In the EMPOWER ADA CEP, we evaluated the impact of PGD on ADA treatment decisions in patients with IBD. Twenty-one HCPs experienced in IBD management, from different types of practices and geographical areas, participated in the CEP. HCPs already incorporated TDM into their practice, suggesting that they recognized the importance of personalized medicine. The experience in IBD and TDM of the HCPs surveyed in this study lends credibility to the data, and could facilitate the use of PGD by less specialized HCPs.

The patient selection for this CEP was at the HCPs’ discretion, resulting in a nearly equal distribution of patients with active disease and patients in remission. Although a selection bias cannot be excluded, this study design allowed for evaluation of the use of the test in the real world. The test was ordered for various clinical scenarios, proactively and reactively, and for patients with different types of IBD, demonstrating its wide applicability. In line with these findings, HCPs ordered the test for cases in remission (PGA = 0) when contemplating dose optimization or de-escalation, and for cases not in remission (PGA > 0) when a loss of response was suspected. The broad utility of the test to inform treatment decisions across a diverse IBD patient population was further emphasized by its use across patients with varying types of IBD (CD, UC, and IC) and with different disease durations and characteristics.

The PGD test enhances the value proposition beyond traditional TDM, by providing CL data and personalized dosing alternatives to guide treatment optimization. CL is emerging as a crucial PK parameter in therapy optimization in IBD, not only for anti-TNFα, but also for other biologics, as recently reviewed by Deyhim at al [41]. Evidence suggests that IFX concentration and CL are superior predictors of clinical and biochemical remission compared to either parameter alone. Furthermore, lower ADA CL is more strongly associated with endoscopic remission, sustained clinical and biochemical remission, and reduced fecal calprotectin levels than ADA concentration alone [26].

The PGD test played a significant role in guiding treatment decisions for patients with IBD. The test results were particularly instrumental in cases where the existing treatment regimen was deemed sufficient to manage IBD symptoms. This was observed in a substantial number of cases, even among those with active disease, where symptoms were reported as mild. The presence of anti-drug antibodies, which can impact treatment efficacy, was minimal in this group. Furthermore, the ADA concentration (median 15 µg/mL) and CL (median 0.268 L/day) levels in this group were noteworthy. The observed concentration exceeded 12 µg/mL, a level linked with endoscopic remission in CD [42]. Furthermore, CL was below the threshold that is associated with improved disease control and a higher probability of achieving endoscopic remission. These factors collectively highlight the importance of considering individual patient characteristics and disease activity when optimizing therapy.

Given the pharmacokinetic differences between CD and UC, ADA dosing strategies often require adjustment. While both conditions follow the same induction regimen (160 mg at week 0, 80 mg at week 2, then 40 mg every two weeks), UC patients frequently require dose intensification (40 mg weekly) due to higher CL, increased fecal ADA loss, and systemic inflammation [38,39]. These factors contribute to subtherapeutic ADA levels, necessitating dose escalation to maintain therapeutic drug exposure [41,42]. Our findings align with these observations, as intensification was more frequent in active disease with high CL, whereas de-escalation was considered in remission cases with high ADA concentrations and low CL.

ADA dosing is further influenced by disease severity. Mild disease cases may remain on 40 mg every two weeks [43], while moderate cases often require 40 mg weekly, especially with low drug levels or high CL [44]. Severe disease activity may need induction, dose escalation, or combination therapy [45]. PGD test results guided dosing decisions, particularly in active disease with high CL, where escalation was necessary, and in remission with high ADA and low CL, where de-escalation minimized overtreatment. This supports PK-driven dosing, incorporating CL and forecasted trough levels to optimize biologic therapy [33,34,41].

In a subset of cases, mainly in remission and with high ADA concentrations and low CL, PGD test results supported the decision to de-escalate ADA treatment. The test results, therefore, appear to reinforce the clinical decision-making process, particularly when considering dose reduction to avoid unnecessary treatment. PGD, by providing individualized PK profiles and CL data, emerges as a valuable tool in this context. Although not assessed in this study, the de-escalation of ADA therapy could potentially lead to cost savings. Further studies are needed to evaluate the economic benefits of PGD in the management of IBD [8].

PGD test results were also taken into consideration to make treatment decisions in cases for whom ADA therapy was either intensified or discontinued. This was observed in a significant number of cases, predominantly among those with PGA > 0. These cases exhibited the lowest ADA concentrations, the highest CL, and the highest rate of anti-drug antibodies, which are factors known to influence treatment outcomes [46]. It is interesting to note that in the intensification group, the decision was made to intensify therapy rather than discontinue it, despite lower ADA concentrations. This could be attributed to the consensus among IBD experts that discontinuation should not be considered until the ADA concentration reaches at least 10–15 µg/mL [37]. While ADA intensification may lead to higher drug costs, appropriate pharmacological treatment can also lead to a reduction in corticosteroid use, hospitalizations, and surgeries [13], ultimately decreasing overall health care costs [47].

Cases for whom ADA was discontinued were treated with compounds with different mechanisms of action, likely because a mechanistic failure was suspected, particularly in cases with higher drug concentrations (>10 µg/mL). Upon a detailed examination of the discontinuation group, we observed that a subset of cases had normal ADA concentrations. Therefore, we conducted a subset analysis, categorizing cases based on whether their ADA concentration was ≤10 or >10 µg/mL. Cases with ADA ≤ 10 µg/mL had higher CL, and half presented with anti-drug antibodies. The higher CL observed in the lower drug concentration subgroup (median 1.158 L/day) compared to the other subgroup (median 0.232 L/day) supports this hypothesis, and suggests faster elimination of the drug from the body, which could lead to subtherapeutic levels. Additionally, the presence of anti-drug antibodies in 50% of cases with lower ADA concentration points to an immunogenic response, which is a known mechanism of biologic therapy failure [13]. Anti-drug antibodies can neutralize the therapeutic effect of ADA, leading to decreased efficacy and the need to switch to another biologic agent. The lack of significant differences in albumin levels and PGA scores between the two subgroups indicates that the overall clinical assessment of disease activity may not capture subtleties in drug levels and CL. This underscores the importance of monitoring drug concentrations and CL to identify potential mechanistic failures early on, and using PGD test results to individualize treatment accordingly.

Logistic regression analysis underscored the importance of CL in ADA therapy decision-making. CL above the cutoff that is considered intermediate (CL ≥ 0.318 L/day) [32] was associated with a 10.4-fold higher likelihood of ADA therapy intensification. In addition, high CL (>0.8 L/day) was significantly associated with both ADA discontinuation and PGA > 0 (active disease), consistent with the findings that high CL is associated with poor outcomes [32]. We confirmed in this study that several factors influence ADA CL in IBD, including disease activity as measured by PGA, albumin concentration, and the presence of anti-drug antibodies [26,27,28]. Incorporating CL in decision-making allows clinicians to tailor treatments, optimize dosages, and indirectly improve outcomes by identifying patients needing adjustments based on their unique CL [41].

According to the HCPs who participated in this CEP, the PGD test was easy to order, the turnaround time was acceptable, and the report was easy to interpret. Not only did the test facilitate therapeutic decision-making, but it was also helpful in providing justification to insurance providers for adjustments of ADA dose and/or interval, and in discussing dose escalation and adverse events with patients. Although current guidelines do not address how such testing should be utilized in clinical practice, in this real-world study, the PGD test provided guidance on the management of patients with IBD treated with ADA.

HCPs noted that an essential aspect that enhances the value of this test is its convenience for both patients and providers. Unlike other tests that require blood collection at precise times, the PGD test eliminates this burden. It can be ordered at any time during the therapy cycle, allowing patients to undergo the test without the need for additional appointments or inconveniences. This streamlines their experience and enables HCPs to implement an individualized therapy plan based on objective results more quickly. For busy clinics, this simplicity translates into improved patient compliance and overall efficiency.

The limitations of this CEP include the absence of a control arm, the small sample size of HCPs and cases, the self-reported nature of therapy changes which could not be verified, and the lack of long-term patient follow-up to evaluate outcomes. While the physician global assessment (PGA) is a subjective measure of disease activity, it remains a valuable tool in IBD [38,39,48], as not all patients have endoscopy or biomarker results available. A retrospective analysis will be conducted in a subsequent study to assess objective patient outcomes and health care costs. Further studies in pediatric patients with IBD will be undertaken to evaluate ADA clearance in this population.

## 5. Conclusions

The PGD test enables personalized treatment of patients with IBD on ADA, by incorporating population PK modeling with Bayesian priors and patient specific clinical and serological inputs to report CL, patient-specific ADA PK profiles, and guidance on alternative dose and/or interval, to achieve treatment targets. In this clinical utility CEP, test results were used by HCPs to guide therapy changes or discontinuation, or to provide reassurance that no changes were necessary. Low drug concentrations and high CL were associated with PGA > 0 (active disease) and ADA intensification or discontinuation, demonstrating that HCPs found utility in these parameters in helping them to implement therapy changes. The ability to perform the test without restrictions on the timing of drug administration enhances its utility in real-world practice and enables precision-guided therapy, which may improve the durability of response.

## Figures and Tables

**Table 1:** Patient characteristics and precision-guided dosing (PGD) test data.

	HCP’s Decision Regarding ADA Therapy					
Characteristic	Overall N = 213 (100%)	Reduction n = 7 (3%)	Continuation n = 127 (60%)	Intensification n = 51 (24%)	Discontinuation n = 28 (13%)	*p* -Value
Age, years	40 (27–56)	31 (25–38)	36 (27–52)	42 (29–56)	48 (31–63)	0.13
Sex, male	109 (51)	3 (43)	65 (51)	25 (49)	16 (57)	0.87
Diagnosis						0.20
CD	162 (76)	5 (72)	98 (77)	41 (80)	18 (64)	
UC	47 (22)	1 (14)	27 (21)	10 (20)	9 (32)	
IC	4 (2)	1 (14)	2 (2)	0 (0)	1 (4)	
Montreal Location for CD						0.78
L1 ileal	46 (29)	3 (60)	27 (28)	11 (28)	5 (28)	
L2 colonic	29 (18)	0 (0)	18 (18)	9 (22)	2 (11)	
L3 ileocolonic	85 (53)	2 (40)	52 (54)	20 (50)	11 (61)	
L4 upper disease	7 (4)	1 (20)	3 (3)	3 (7)	0 (0)	
Montreal Behavior for CD						0.20
B1 non-stricturing, non-penetrating	80 (51)	2 (40)	52 (54)	21 (52)	5 (31)	
B2 stricturing	56 (36)	1 (20)	32 (34)	13 (33)	10 (63)	
B3 penetrating	20 (13)	2 (40)	11 (12)	6 (15)	1 (6)	
p perianal disease	30 (19)	0 (0)	15 (15)	7 (17)	8 (44)	
Montreal Extent for UC						0.47
E1 rectum	3 (6)	0 (0)	2 (8)	1 (10)	0 (0)	
E2 left sided	17 (36)	0 (0)	7 (27)	3 (30)	6 (67)	
E3 extensive	27 (58)	1 (100)	17 (65)	6 (60)	3 (33)	
Disease duration, years						0.31
Less than 1	4 (2)	0 (0)	1 (1)	2 (4)	1 (4)	
1 to 5	55 (26)	0 (0)	37 (29)	12 (24)	6 (21)	
More than 5	154 (72)	7 (100)	89 (70)	37 (73)	21 (75)	
Concomitant therapies						0.06
Immunomodulator	31 (15)	2 (29)	16 (13)	9 (18)	4 (14)	
Corticosteroid	9 (4)	0 (0)	2 (2)	3 (6)	4 (14)	
Active disease, PGA > 0 ^a^	114 (54)	0 (0)	45 (35)	44 (88)	25 (89)	**<0.001**
Forecasted ADA trough, µg/mL	10 (7–16)	21 (10–25)	14 (10–18)	6 (4–8)	10 (5–13)	**<0.001**
Measured ADA, µg/mL	12 (7–17)	21 (16–25)	15 (10–18)	6 (4–8)	10 (4–14)	**<0.001**
Measured ADA > 10, µg/mL	129 (61)	6 (86)	101 (80)	6 (12)	16 (57)	**<0.001**
Albumin, g/dL	3.9 (3.7–4.3)	4.1 (3.9–4.4)	4.0 (3.7–4.3)	3.8 (3.6–4.0)	3.9 (3.7–4.1)	**0.02**
ATA presence, >1.7 U/mL	14 (7)	0 (0)	3 (2)	5 (10)	6 (21)	**0.003**
Clearance, L/day	0.334 (0.214–0.488)	0.272 (0.229–0.344)	0.268 (0.192–0.380)	0.496 (0.372–0.865)	0.292 (0.228–0.840)	**<0.001**
Clearance < 0.318, L/day	100 (47)	5 (71)	74 (58)	6 (12)	15 (54)	**<0.001**

Values are presented as n (%) or median (IQR). Statistical analysis consisted of Kruskal–Wallis rank sum test for continuous variables and Fisher’s exact test for categorical variables. Bolded *p*-values (*p* < 0.05) indicate statistically significant differences between treatment decision groups (reduction, continuation, intensification, discontinuation), identifying factors associated with ADA therapy modifications. ^a^ Disease was considered active if HCPs indicated mild, moderate, or severe symptoms in PGA. For one case in intensification group, PGA was not provided. ADA: adalimumab or its biosimilars; ATA: anti-drug antibodies to adalimumab; CD: Crohn’s disease; HCP: health care provider; IC: indeterminate colitis; IQR: interquartile range; PGA: physician global assessment; UC: ulcerative colitis.

**Table 2:** Univariate logistic regression analysis of pharmacokinetic predictors associated with adalimumab (ADA) therapy changes and active disease.

Characteristic	Cutoff	OR (95% CI)	*p* -Value
**Measured ADA concentration, μg/mL**			
ADA therapy intensification	≤10	23.7 (10.0 to 65.7)	**<0.001**
ADA therapy discontinuation	<5	3.30 (1.23 to 8.30)	**0.019**
PGA > 0	≤10	3.02 (1.70 to 5.49)	**<0.001**
**ADA Clearance, L/day**			
ADA therapy intensification	≥0.318	10.40 (4.48 to 28.30)	**<0.001**
ADA therapy discontinuation	>0.8	3.49 (1.30 to 8.84)	**0.014**
PGA > 0	>0.8	4.20 (1.63 to 13.0)	**0.002**

Each row represents separate model. Dependent variables were binary outcomes (yes/no) for ADA therapy intensification, discontinuation, or active disease (PGA > 0). OR with 95% CI and *p*-values indicate strength and significance of associations. ADA: adalimumab or its biosimilars; CI: confidence interval; OR: odds ratio; PGA: physician global assessment. *p*-values < 0.05 are bolded.

**Table 3:** Multivariate logistic regression analysis of clinical factors associated with high clearance (>0.8 L/day).

Characteristic	OR (95% CI)	*p* -Value	OR (95% CI)	*p* -Value
Disease activity (PGA = 0 vs. PGA > 0)	5.53 (1.53 to 27.4)	**0.008**	5.26 (1.53 to 25.0)	**0.007**
ATA (≥1.7 vs. <1.7 U/mL)	138 (23.0 to 1474)	**<0.001**	127 (23.7 to 1149)	**<0.001**
Albumin (<4 vs. ≥4 g/dL)	7.08 (1.76 to 41.3)	**0.005**	6.77 (1.92 to 33.3)	**0.002**
Sex (female vs. male)	0.75 (0.22 to 2.50)	0.63		
Age (≤40 vs. >40 years)	1.94 (0.59 to 7.16)	0.28		
Diagnosis (CD vs. UC)	0.71 (0.10 to 5.59)	0.73		
Montreal Location (L1, L2 vs. L3)	4.98 (1.16 to 28.0)	**0.030**	3.50 (1.16 to 11.6)	**0.026**
Montreal Behavior (B1 vs. B2, B3)	0.89 (0.20 to 3.92)	0.87		
Montreal Behavior (perianal vs. non-perianal)	0.74 (0.15 to 3.16)	0.69		
Disease duration (≤5 vs. >5 years)	1.36 (0.32 to 7.66)	0.69		
Concurrent immunomodulator or steroid therapy (ADA monotherapy vs. combination therapy)	2.36 (0.52 to 9.94)	0.25		

Multivariate analysis was conducted with backward elimination of factors with *p* > 0.05. Last two columns present final model obtained through backward elimination, including only significant predictors. Cases with indeterminate colitis (n = 4) were excluded from this analysis. ADA: adalimumab or its biosimilars; ATA: anti-drug antibodies to ADA; CD: Crohn’s disease; CI: confidence interval; OR: odds ratio; UC: ulcerative colitis. *p*-values < 0.05 are bolded.

## Data Availability

The original contributions presented in this study are included in the article/supplementary material. Further inquiries can be directed to the corresponding author.

## Supplementary Materials

The following supporting information can be downloaded at https://www.mdpi.com/article/10.3390/pharmaceutics17040428/s1: Figure S1: Comparison between forecasted ADA trough concentrations and measured ADA concentrations; Figure S2: Reasons for using therapeutic drug monitoring (TDM) for adalimumab (ADA) based on pre-program survey; Figure S3: Other therapy changes and ordering of imaging and laboratory tests; Table S1: Pre-program survey results from participating health care providers (HCPs, n = 21); Table S2: Post-program survey results from participating health care providers (HCPs, n = 21); Table S3: Therapy changes and ordering of imaging and laboratory tests.

## Abbreviations

The following abbreviations are used in this manuscript:

ADA	Adalimumab or its biosimilars
AE	Adverse event
ATA	Anti-drug antibodies to adalimumab
CD	Crohn’s disease
CEP	Clinical experience program
Cl	Clearance
EMPOWER	Effect on Decision-Making of Precision Optimization in Real-World Evidence Research—Adalimumab
HCP	Health care provider
IC	Indeterminate colitis
IQR	Interquartile range
MIPD	Model-informed precision dosing
OR	Odds ratio
PGA	Physician global assessment of disease activity
PGD	Precision-guided dosing
PK	Pharmacokinetics
PopPK	Population pharmacokinetics
TDM	Therapeutic drug monitoring
UC	Ulcerative colitis
