# Supplementary material for: The Clinical Utility of Precision-Guided Dosing for Adalimumab Therapy Optimization in Inflammatory Bowel Disease: A Clinical Experience Program
Source: Pharmaceutics. 2025 Mar 27;17(4):428. doi: 10.3390/pharmaceutics17040428 (PMC12030419; doi:10.3390/pharmaceutics17040428)
Supplement: Supplementary file 1 [file pharmaceutics-17-00428-s001.zip › pharmaceutics-3522890-supplementary.pdf]

pharmaceutics

MDPI

The Clinical Utility of Precision-Guided Dosing for Adalimumab Therapy Optimization in Inflammatory Bowel Disease: A Clinical Experience Program

Supplementary data

Supplementary Figures

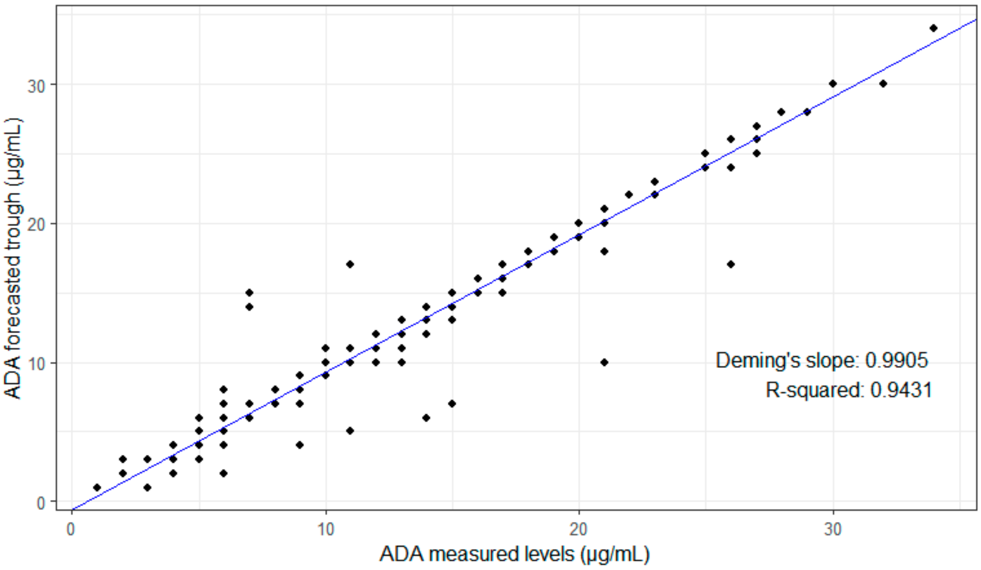

Figure S1. Comparison between forecasted ADA trough concentrations and measured ADA concentrations. ADA: adalimumab or its biosimilars.

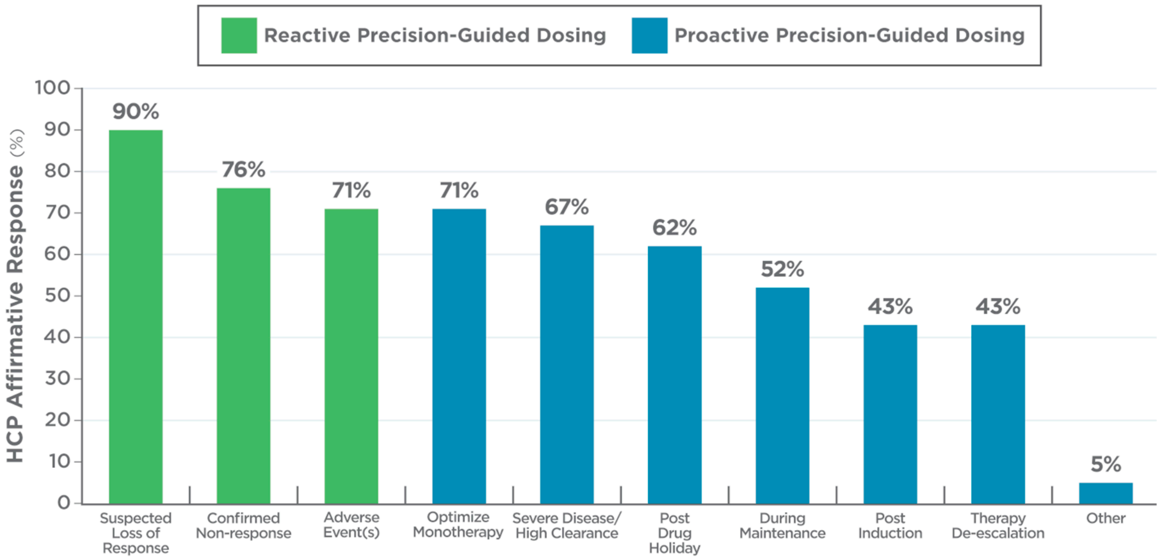

Figure S2. Reasons for using therapeutic drug monitoring (TDM) for adalimumab (ADA) based on pre-program survey.

Bars report percentage of health care providers (HCPs, n = 21) who indicated using reactive or proactive therapeutic drug monitoring (TDM) for reasons listed in graph legend on left. HCPs could select multiple reasons. Thus, total exceeds 100%. ADA: adalimumab or its biosimilars; CL: clearance; TDM: therapeutic drug monitoring.

pharmaceutics

MDPI

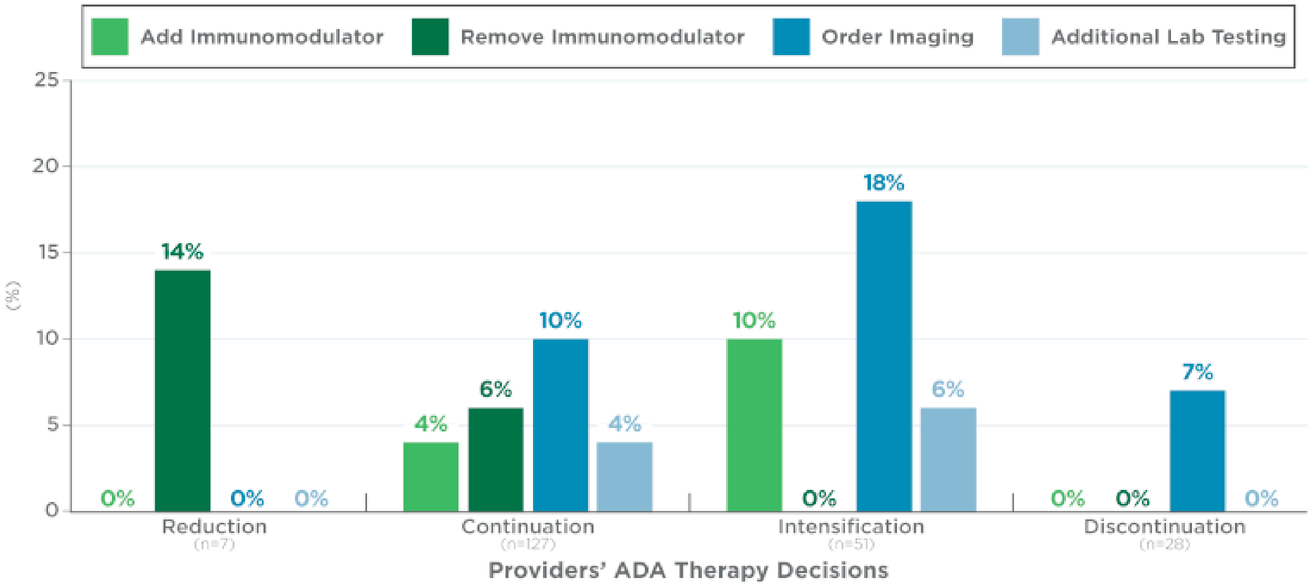

Figure S3. Other therapy changes and ordering of imaging and laboratory tests.

Percentage of cases (N = 213) for whom HCPs reported adjustments to alternative treatments and scheduling of diagnostic imaging and laboratory evaluations in each group.

pharmaceutics

MDPI

Table S1. Pre-program survey results from participating health care providers (HCPs, n = 21).

**Question/Topic for participating HCP**		n (%)
Number of years in practice		
	< 5	1 (4.8)
	5 to 10	2 (9.5)
	> 10	18 (85.7)
Monthly patients with IBD[a]		
	10 to 25	1 (4.8)
	25 to 50	6 (28.6)
	> 50	14 (66.7)
Patients with CD vs. UC		
	higher percentage of CD	6 (28.6)
	higher percentage of UC	3 (14.3)
	roughly equal percentage	12 (57.1)
Percentage on ADA/ADA biosimilar		
	< 10%	2 (9.5)
	10% to 20%	14 (66.7)
	21% to 40%	4 (19.0)
	> 40%	1 (4.8)
ADA/ADA biosimilar + immuno-suppressant		
	almost never (< 10%)	1 (4.8)
	infrequently (10-50%)	12 (57.1)
	frequently (> 60-90%)	6 (28.6)
	most of the time (> 90%)	2 (9.5)
For a patient who is losing response to ADA monotherapy (secondary loss of response), what would you consider (rank steps, from 1 to 6, based on the most frequent scenarios):		
Perform TDM		
	1	21 (100)
Increase the dose		
	2	3 (14.3)
	3	5 (23.8)
	4	5 (23.8)
	6	8 (38.1)
Decrease frequency		
	2	15 (71.4)
	3	5 (23.8)
	5	1 (4.8)
Add immunosuppressant		

Question/Topic for participating HCP		n (%)
	2	1 (4.8)
	3	8 (38.1)
	4	7 (33.3)
	5	2 (9.5)
	6	3 (14.3)
Switch to a different anti-TNFα[a]		
	2	1 (4.8)
	3	3 (14.3)
	4	5 (23.8)
	5	9 (42.9)
	6	3 (14.3)
Switch to a different mechanism of action		
	2	1 (4.8)
	4	4 (19.0)
	5	9 (42.9)
	6	7 (33.3)
Target a specific ADA level		
	Yes	15 (71.4)
	No	6 (28.6)
If yes, target level (mg/ml)[a]		
	10 -15	1 (6.7)
	>12	1 (6.7)
	>10	1 (6.7)
	10	4 (26.7)
	>8	1 (6.7)
	8	2 (13.3)
	>7.5	2 (13.3)
	7	1 (6.7)
	5	1 (6.7)
	As recommended by Prometheus	1 (6.7)
Scenarios of when and how ADA TDM is used in clinical practice [b]:		
Reactive TDM for confirmed primary or secondary nonresponse		
Reactive TDM for suspicion of loss of response		16 (76.2)
Proactive TDM after induction		19 (90.5)

pharmaceutics MDPI

Question/Topic for participating HCP	n (%)
Proactive TDM during maintenance	9 (42.9)
Proactive TDM in severe patients or patients with high CL	11 (52.4)
Proactive TDM to optimize ADA monotherapy	14 (66.7)
Proactive TDM to de-escalate	15 (71.4)
Proactive TDM after a drug holiday	9 (42.9)
TDM due to adverse events	13 (61.9)
Other reasons for TDM	15 (71.4)
	1 (4.8)

[a] Due to rounding, total percentage does not precisely equal 100. [b] Proactive therapeutic drug monitoring (TDM) after induction usually refers to TDM after second week of adalimumab (ADA) treatment, whereas proactive TDM during maintenance usually refers to TDM after fourth dose of ADA, corresponding to eighth week of treatment.

pharmaceutics

MDPI

Table S2. Post-program survey results from participating health care providers (HCPs, n = 21).

Question	Response Options	# Responses n (%)
Which of the following best describes your experience with PredictrPK ADA	a. The test has high value in providing precision-guided adalimumab dosing	21 (100)
	b. The test has limited value in providing precision-guided adalimumab dosing	0 (0)
Was PredictrPK ADA easy to order?	Yes	18 (86)
	No	3 (14)
Were the results received back in a reasonable amount of time?	Yes	20 (95)
	No	1 (5)
Was the test report easy to understand and informative?	Yes	21 (100)
	No	0 (0)
After having used PredictrPK ADA, what scenarios do you think is the test most useful? *(Check all that apply)*	a. There is no added value from PredictrPK ADA	0 (0)
	b. Patients starting adalimumab therapy (post-induction or first maintenance dose)	8 (38)
	c. Patients on maintenance adalimumab therapy	13 (62)
	d. Patients experiencing primary loss of response	18 (86)
	d. Patients experiencing secondary loss of response	18 (86)
	e. When therapy escalation is being considered	20 (95)
	f. When therapy de-escalation is being considered	16 (76)
	g. Patients experiencing injection reactions	6 (29)
	h. Patients experiencing other adverse events, i.e., recurrent infections, etc.	8 (38)
	i. Other scenarios	0 (0)
Having used the PredictrPK ADA test, do you believe that the results of the test were beneficial in the ongoing treatment of your patients?	Yes	21 (100)
	No	0 (0)
If yes, how?	a. Assisted in deciding the optimal dose and/or intervals	17 (81)
	b. Assisted in providing confidence in the ongoing dose and/or interval	14 (67)
	c. Assisted in discussions with patients when they required a dose escalation	17 (81)

Question	Response Options	# Responses n (%)
	d. Assisted in discussions with patients regarding adverse events	6 (29)
	e. Convincing insurance provider of need for dose or interval change	10 (48)
	f. Other reasons	0 (0)
Do you believe that the PredictrPK ADA (Precision-guided dosing) could add more value to your clinical practice than standard TDM (drug level only)?	Yes	21 (100)
	No	0 (0)
If yes, how?	a. The test will allow proactive ADA dose optimization at post-induction	14 (67)
	b. The test will help determine early during therapy if ADA is the right therapy for the patient	8 (38)
	c. The test will help switch to a proactive TDM strategy after having used a reactive TDM strategy in the past	11 (52)
	d. The test will allow proactive ADA dose optimization and monitoring during the maintenance phase to achieve and/or sustain the desired clinical outcome	16 (76)
	e. The test will allow determination of the right ADA dose and/or interval in patients with severe disease where it may be challenging to maintain the serum drug levels above a certain desired concentration simply by empiric dose escalation	16 (76)
	f. The test will aid in ADA dose de-escalation if being considered in patients in remission	13 (62)
	g. The test will help in identifying the right dose and/or interval in patients restarting ADA after a drug holiday	11 (52)
	h. The test will help in optimizing ADA monotherapy in patients where removal of concomitant immunotherapy is being considered	16 (76)
	i. The test will help in identifying patients where ADA therapy should be discontinued	16 (76)
	j. The test will help explain tolerance and/or adverse events that the patients may be experiencing due to ADA	7 (33)
	k. Predict PK test adds value in other scenarios	1 (5)

Question	Response Options	# Responses n (%)
On a scale of 1-10 how likely would you utilize PredictrPK ADA in regular practice (1 being least likely, 10 being most likely)	10	7 (33)
	9	5 (24)
	8	5 (24)
	7	4 (19)

PredictrPK ADA: commercial name of PGD test.

pharmaceutics

MDPI

Table S3. Therapy changes and ordering of imaging and laboratory tests.

Characteristic, n (%)	Overall N = 213	Reduction n = 7	Continuation n = 127	Intensification n = 51	Discontinuation n = 28
Discontinue IFX therapy	28 (13)	0 (0)	0 (0)	0 (0)	28 (13)
Switch to:					
RZB	4 (1.9)				4 (1.9)
UPA	6 (2.8)				6 (2.8)
UST	9 (4.2)				9 (4.2)
Other	8 (3.8)				8 (3.8)[a]
N/A	1 (0)				1 (0)
Add immunomodulator	10 (5)	0 (0)	5 (4)	5 (10)	0 (0)
Remove immunomodulator	9 (4)	1 (14)	8 (6)	0 (0)	0 (0)
Order imaging test	23 (11)	0 (0)	12 (10)	9 (18)	2 (7)
Order laboratory test	8 (4)	0 (0)	5 (4)	3 (6)	0 (0)

[a]: For the 8 cases reported as "other", the health care providers (HCPs) noted that treatment decision was pending discussion with the patient. IFX: Infliximab; N/A: Not available; RZB: Risankizumab; UPA: Upadacitinib; UST: Ustekinumab.
